# Alternative Pathway Is Involved in Hydrogen Peroxide-Enhanced Cadmium Tolerance in Hulless Barley Roots

**DOI:** 10.3390/plants10112329

**Published:** 2021-10-28

**Authors:** Li He, Xiaomin Wang, Xiaofan Na, Ruijun Feng, Qiang He, Shengwang Wang, Cuifang Liang, Lili Yan, Libin Zhou, Yurong Bi

**Affiliations:** 1 State Key Laboratory of Hulless Barley and Yak Germplasm Resources and Genetic Improvement, Lhasa 850002, China; hel16@lzu.edu.cn (L.H.); wangxiaomin@lzu.edu.cn (X.W.); naxf@lzu.edu.cn (X.N.); 2 Ministry of Education Key Laboratory of Cell Activities and Stress Adaptations, School of Life Sciences, Lanzhou University, Lanzhou 730000, China; fengrj16@lzu.edu.cn (R.F.); hjtywjb@163.com (Q.H.); wangshw16@lzu.edu.cn (S.W.); liangcf16@lzu.edu.cn (C.L.); yanll18@lzu.edu.cn (L.Y.); 3 School of Biological and Pharmaceutical Engineering, Lanzhou Jiaotong University, Lanzhou 730070, China; 4 Biophysics Group, Institute of Modern Physics, Chinese Academy of Sciences, Lanzhou 730000, China

**Keywords:** alternative pathway, Cd stress, hulless barley, hydrogen peroxide, reactive oxygen species

## Abstract

Hulless barley, grown in the Qinghai Tibet Plateau, has a wide range of environmental stress tolerance. Alternative pathway (AP) and hydrogen peroxide (H_2_O_2_) are involved in enhancing plant tolerance to environmental stresses. However, the relationship between H_2_O_2_ and AP in hulless barley tolerance to cadmium (Cd) stress remains unclear. In the study, the role and relationship of AP and H_2_O_2_ under Cd stress were investigated in hulless barley (Kunlun14) and common barley (Ganpi6). Results showed that the expression level of alternative oxidase (AOX) genes (mainly *AOX1a*), AP capacity (V_alt_), and AOX protein were clearly induced more in Kunlun14 than in Ganpi 6 under Cd stress; moreover, these parameters were further enhanced by applying H_2_O_2_. Malondialdehyde (MDA) content, electrolyte leakage (EL) and NAD(P)H to NAD(P) ratio also increased in Cd-treated roots, especially in Kunlun 14, which can be markedly alleviated by exogenous H_2_O_2_. However, this mitigating effect was aggravated by salicylhydroxamic acid (SHAM, an AOX inhibitor), suggesting AP contributes to the H_2_O_2_-enhanced Cd tolerance. Further study demonstrated that the effect of SHAM on the antioxidant enzymes and antioxidants was minimal. Taken together, hulless barley has higher tolerance to Cd than common barley; and in the process, AP exerts an indispensable function in the H_2_O_2_-enhanced Cd tolerance. AP is mainly responsible for the decrease of ROS levels by dissipating excess reducing equivalents.

## 1. Introduction

Cadmium (Cd), the third major contaminant to the environments, is seriously harmful to organisms, even human health [1,2]. Cd can affect plant growth and development, such as yellowing of leaves, necrosis of roots, inhibition of photosynthesis, changes of transpiration and respiration rate [3,4]. Moreover, Cd can damage DNA and change protein structure [5]. In cells, the over-accumulated Cd can disrupt the redox homoeostasis and further result in oxidative stresses [6]. In order to reduce the toxicity of Cd, plants have developed a variety of defense mechanism [7].

Plants can suppress Cd uptake to maintain a low Cd concentration, thus avoiding heavy metal toxicity [8]. Meanwhile, chelating and sequestrating Cd to insensitive compartments of cells (e.g., vacuoles) was verified to be momentous in Cd detoxification [9]. The activation of antioxidant defense system has been widely proven to be an essential way to resist Cd-induced oxidative stress [10]. A series of heavy metal transport-associated proteins, such as yellow stripe-like protein (YSL), natural resistance-associated macrophage protein (NRAMP), and heavy metal transporting ATPase (HMA), transport heavy metal ions to outside of the cytoplasm, thus maintaining the intracellular ion homeostasis and enhancing Cd tolerance [11]. In addition, alternative pathway (AP) has been extensively reported to enhance Cd tolerance [8]. Even so much previous effort has been made, the in-depth protective mechanisms in plant tolerance to Cd stress is still unclear.

H_2_O_2_ signal is widely involved in the plant responses to biotic and abiotic stresses [12]. Only high-concentration H_2_O_2_ can lead to serious oxidative damages. Under aluminum (Al) stress, H_2_O_2_ can obviously recover the Al-induced root growth inhibition and reduce Al accumulation in roots through improving antioxidant enzyme activities and gene expression in peanut roots [10]. H_2_O_2_ regained crop development and subsequent activation of MPK1/2 by enhancing the activities of antioxidant enzymes and the content of AsA and GSH under Cd tolerance in *Solanum lycopersicum* [13]. Moreover, H_2_O_2_ was found to improve thiol content, antioxidant enzyme activities, activation of metallothionein protein (*BnMP1*) mRNA and decrease lipid peroxidation in Brassica napus exposed to chromium (Cr) stress [14]. H_2_O_2_ was also reported to be involved in signal perception and transduction of cold stress in *Synechocystis* [15]. Meanwhile, H_2_O_2_ protects bacteria from oxidative stress via modulating the activity of transcription factors *OxyR* and *PerR* [16]. In addition, H_2_O_2_ regulates the ethylene signal in response to the hypoxic stress [17]. These results confirm that a suitable H_2_O_2_ concentration can strengthen the tolerance of plants to environmental stresses. However, it is still unclear about the role of H_2_O_2_ in hulless barley tolerance to Cd stress. 

Respiration metabolism plays fundamental functions in plant growth and development. Plant mitochondria have an alternative pathway (AP) in addition to the cyanide-sensitive cytochrome pathway (CP) [18]. Alternative oxidase (AOX) is the terminal oxidase of AP, and is located in the mitochondria inner membrane [18]. When plants are exposed to environmental stresses, the AP capacity can be significantly increased [19]. In hulless barley, AP capacity and AOX protein level were markedly increased under low-nitrogen stress [20], and exposure to UV-B radiation [21]. It was reported that Cd stress significantly inhibited the CP capacity, but induced the AP capacity in *Euglena* [22]. In Arabidopsis, AP capacity and AOX protein level were also increased under Cd stress [23]. It was reported that H_2_O_2_ is involved in regulating the transcription of *AOX* family genes in *M. grisea* [24]. In addition, H_2_O_2_ induced AP in chill and salt stress [25,26]. However, the relationship and the mechanism between AP and H_2_O_2_ in the highland barley tolerance to Cd stress are still unknown. 

Hulless barley is an ideal material to explore the mechanism of crop tolerance because it grows in such harsh climate conditions [21]. In this study, we explored the role of H_2_O_2_ and AP in hulless barley response to Cd stress. The results showed that AP is involved in H_2_O_2_-enhanced Cd tolerance in hulless barley by dissipating excess reducing equivalents.

## 2. Materials and Methods

### 2.1. Plant Materials and Growth Conditions

Hulless barley (Kunlun14) and common barley (Ganpi6) were provided by Prof. Kunlun Wu (Qinghai Academy of Agriculture and Forestry Sciences, Xi′ning, China). The seeds were treated with 2% NaClO for 10 min, and washed with sterile water for at least 3 times. Then the seeds were germinated and grown in 200 mL plastic beakers filled with 1/4-strengh Hoagland culture solution [27]. Culture solution was changed every other day. 

After 6 d growth, seedlings were used for treatments. 150 μM CdCl_2_ was added in the 1/4 Hoagland solution for 48 h as the Cd stress. 150 μM salicylhydroxamic acid (SHAM) was used to inhibit the alternative oxidase (AOX) activity. A total of 20 μM hydrogen peroxide (H_2_O_2_) was added in solution for 48 h. Roots were collected for the following experiments. 

### 2.2. H_2_O_2_ Staining

H_2_O_2_ staining was performed following the method described by Skórzyńska et al. [28]. Roots were stained in 2 mg/mL 3,3-diaminobenzidine (DAB) solution for 10 h, and photographed using the Leica SM IRBE stereomicroscope (Leica Microsystems, Wetzlar, Germany).

### 2.3. Determination of Electrolyte Leakage and Malondialdehyde Content 

Electrolyte leakage (EL) and malondialdehyde (MDA) content in roots were determined according to the method described by Janicka et al. [29]. 

### 2.4. Measurements of Respiratory Rates

Respiratory rate was detected as described by Wang et al. [30]. 0.05 g of roots were cut into small segments, and then put into the reaction vessel containing 2 mL phosphate buffer (pH 6.8). After reaction for 2 min, oxygen consumption rate was measured, and this rate was defined as the total respiratory rate (V_t_). After 2 mM KCN or 2 mM salicylhydroxamic acid (SHAM) was added into the reaction vessel for 2 min; the oxygen consumption rate was defined as the alternative pathway capacity (V_alt_) or the cytochrome pathway capacity (V_cyt_), respectively. 

### 2.5. Determination of Antioxidant Contents 

Total ascorbic acid (AsA) content, reduced AsA and oxidized AsA were measured according to the method described by Paradiso et al. [27]. Oxidized glutathione (GSSG) and reduced glutathione (GSH) contents were measured according to the method described by Paradiso et al. [27].

### 2.6. Antioxidant Enzyme Activity Assay

The enzymes were extracted according to the method of Pinto et al. [28]. Antioxidant enzyme activities (SOD, CAT, POD and APX) were analyzed following the method described by Jian et al. [29]. The activities of the GSH-AsA cycle-related enzymes (DHAR, MDHAR, GR and GPX) were determined according to the method described by Zhang et al. [4].

### 2.7. RNA Isolation and qRT-PCR

RNA isolation and qRT-PCR were carried out according to the method of He et al. [30]. The gene-specific primers were listed in Table 1. *HvACTIN* was used as the reference gene. qRT-PCR data were quantified using the 2^−^^△△^^C^^T^ method.

### 2.8. Western-Blot Analysis

Western-blot analysis was conducted following the method described by Zhao et al. [21]. Proteins were separated on 12.5% acrylamide gel, then transferred to polyvinylidene difluoride membrane. The membrane was blocked for 3 h with 10% bovine serum albumin. Primary antibody against Arabidopsis AOX was added and incubated overnight. After rinsing three times with TTBS [15 mM NaCl, 0.05% Tween-20, 1 mM Tris-HCl (pH 8.0)], secondary antibody was added and incubated for visualization according to the instructions of the luminescence kit (NCM BIotech; Suzhou; China).

### 2.9. Statistical Analysis

Each experiment was repeated at least three times. The date was analyzed by SPSS 17.0 and Origin 8. Different lowercase letters indicate significant difference at *p* < 0.05.

## 3. Results

### 3.1. Effects of Cd Stress on H_2_O_2_ Content

**Figure 1:**
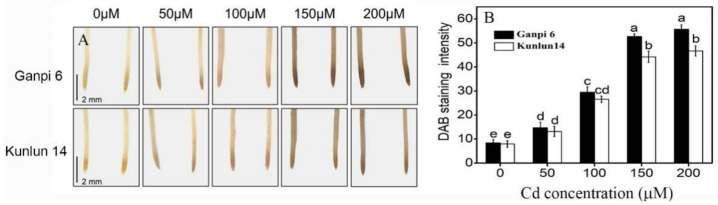
Effects of Cd on H_2_O_2_ content in Ganpi6 and Kunlun14 roots. (**A**) Histochemical staining of H_2_O_2_; (**B**) quantification of H_2_O_2_ content. Six-day-old seedlings were grown in 1/4-strengh Hoagland nutrient solution with 0–200 μM Cd for 48 h. H_2_O_2_ level was examined by histochemical method. Different lower case letters represent significant difference at *p* < 0.05.

To explore whether H_2_O_2_ is involved in enhancing hulless barley tolerance to Cd stress, the H_2_O_2_ content was analyzed under Cd Stress by 3,3-diaminobenzidine (DAB) histochemical staining. The optimum Cd concentration (150 μM) for barley was selected in our previous research [30]. As shown in Figure 1, with the increase of Cd concentration, the H_2_O_2_ staining was gradually deepened in Ganpi6 and Kunlun14 roots. Under 150 μM Cd, H_2_O_2_ staining was increased by 5.26× and 4.18× in Ganpi6 and Kunlun14 roots, respectively. When Cd concentration was increased to 200 μM, the H_2_O_2_ staining was no longer increased. These results indicated that Cd stress can significantly induce H_2_O_2_ accumulation, which was significantly lower in Kunlun14 than that in Ganpi6, suggesting that Ganpi6 suffered more oxidative stress in comparison with Kunlun 14 under Cd stress.

**Figure 2:**
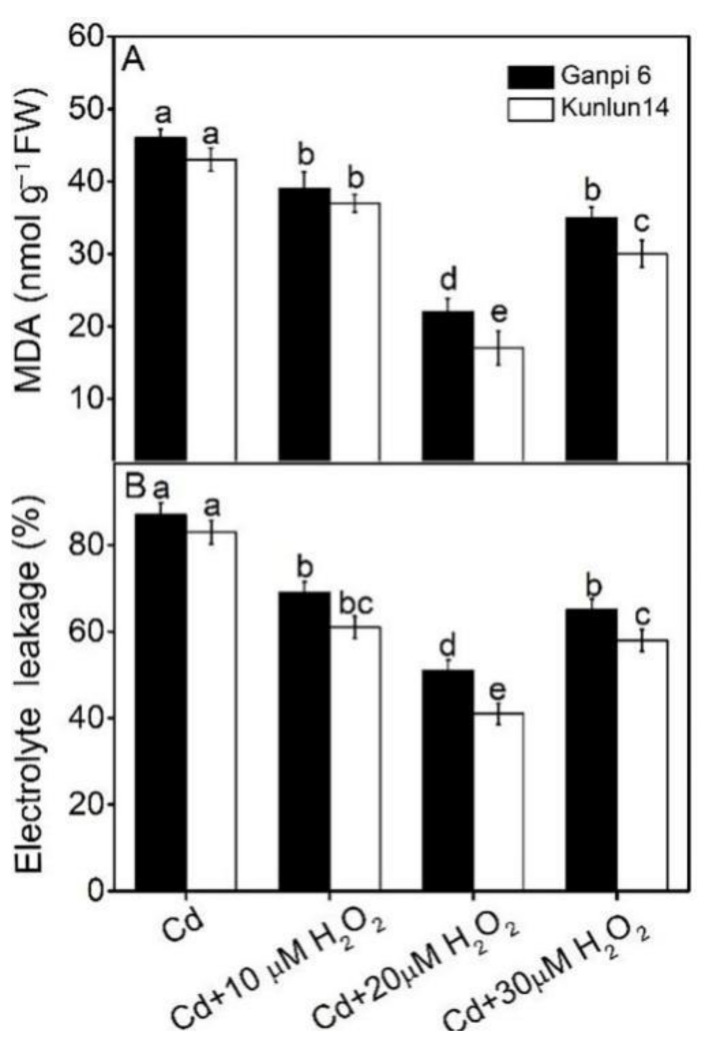
Effects of H_2_O_2_ on malondialdehyde content (MDA) (**A**) and electrolyte leakage (EL) (**B**) under Cd stress in Ganpi6 and Kunlun14 roots. Six-day-old seedlings were grown in 1/4-strengh Hoagland solution with 150 μM Cd and 10–30 μM H_2_O_2_ for 48 h (*n* = 3). Different lower case letters represent significant difference at *p* < 0.05.

To analyze whether H_2_O_2_ has protective effects on Ganpi6 and Kunlun14 roots under Cd stress, exogenous H_2_O_2_ (10, 20 and 30 μM) were applied under 150 μM Cd treatment. H_2_O_2_ function was evaluated by measuring the MDA content and EL level. As shown in Figure 2, after 150 μM Cd + H_2_O_2_ treatment for 48 h, 20 μM H_2_O_2_ significantly reduced the MDA content and EL level in Ganpi6 and Kunlun14 roots. However, when H_2_O_2_ concentration was increased to 30 μM, the MDA content and EL level gradually increased to the level of 10 μM H_2_O_2_ treatment. These results suggested that 20 μM of H_2_O_2_ has the best effect on alleviating the Cd-induced oxidative stress. So 20 μM H_2_O_2_ was used in the further study. In addition, upon Cd + 20 μM H_2_O_2_ treatment, the oxidative stress in Kunlun 14 was significantly lower than that in Ganpi6, further confirming that Kunlun 14 can better tolerate Cd stress.

### 3.2. Exogenous H_2_O_2_ Enhances HvAOXs Expression in Ganpi6 and Kunlun14 Roots under Cd Stress

**Figure 3:**
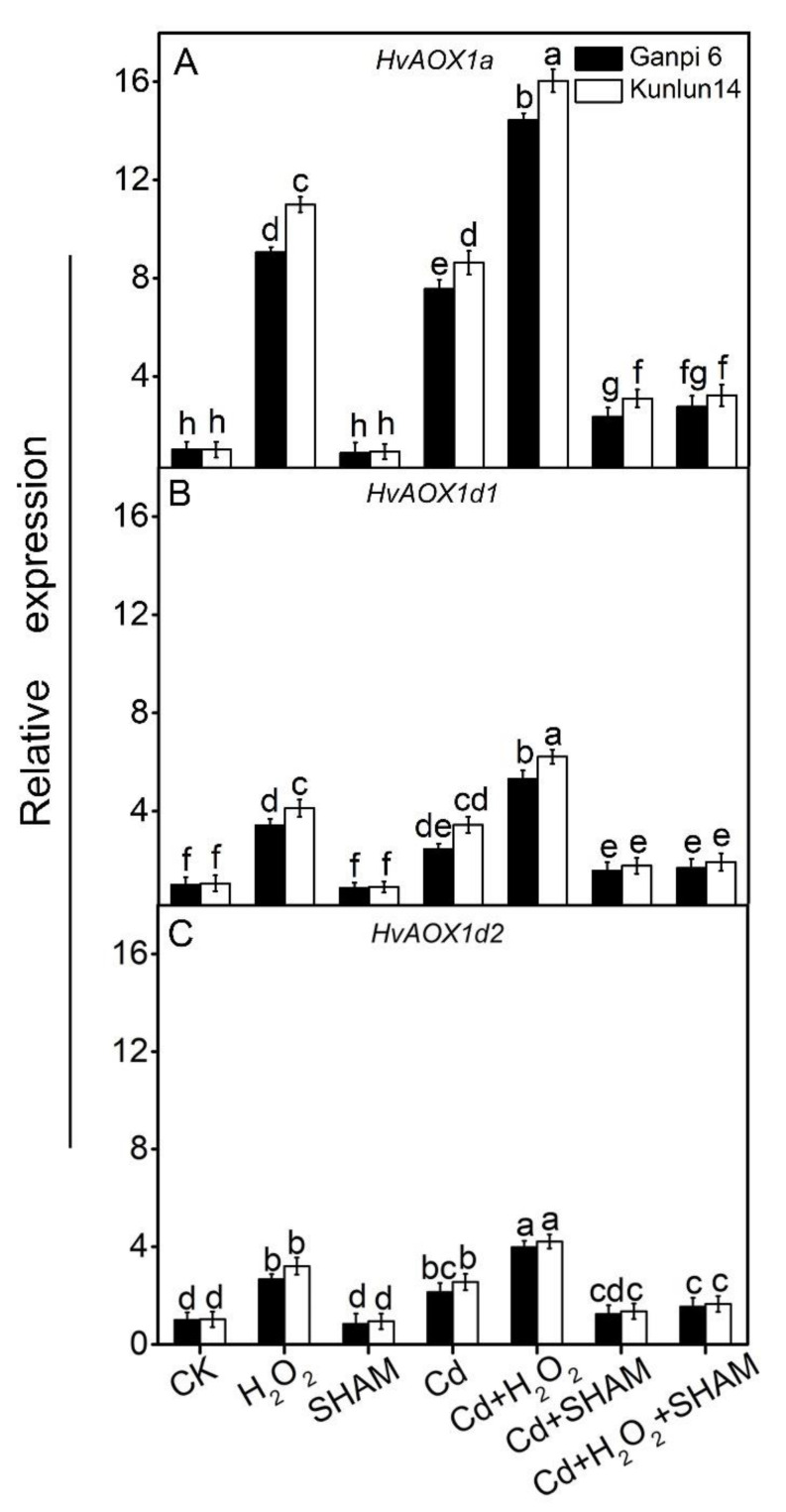
Effects of H_2_O_2_ on the expression of *HvAOXs* genes in Ganpi6 and Kunlun14 roots under Cd stress. (**A**) *HvAOX1a*; (**B**) *HvAOX1d1*; (**C**) *HvAOX1d2*. In this experiment, CK represented seedlings that grown normally without any treatment. 150 μM Cd, 20 μM H_2_O_2_, and 100 μM salicylhydroxamic acid (SHAM) were used. *HvACTIN* was used as the reference gene (*n* = 3).Different lower case letters represent significant difference at *p* < 0.05.

Alternative pathway (AP) can be markedly induced when plants are exposed to various stresses [31]. Under stress conditions, H_2_O_2_ can significantly induce *AOXs* expression and further promote AP capacity [32,33]. By far, *HvAOXs* had been cloned in our previous research [31]. The effect of Cd stress on *HvAOXs* expression was investigated. *HvAOXs* expression was up-regulated in Ganpi6 and Kunlun14 roots under Cd stress. In Ganpi6 and Kunlun14, the expression of *HvAOX1a*, *HvAOX1d1* and *HvAOX1d2* increased by 6.17× and 6.98×, by 1.41× and 1.87×, and by 86.04% and 105.13%, respectively (Figure 3). Under Cd + SHAM treatment, the expression level of *HvAOXs* was observably lowered. The effect of exogenous H_2_O_2_ on *HvAOXs* expression was further investigated. Under Cd + H_2_O_2_ treatment, the expression of *HvAOX1a* was increased by 4.21× and 5.37× in Ganpi6 and Kunlun14 roots, respectively (Figure 3A), whereas *HvAOX1d1* and *HvAOX1d2* were just slightly induced (Figure 3B,C). When AP was inhibited by SHAM, the expression of *HvAOXs* was markedly reduced under Cd + H_2_O_2_ + SHAM treatment (Figure 3). These results suggested that AP might play a crucial role in H_2_O_2_-promoted Cd tolerance.

### 3.3. Exogenous H_2_O_2_ Enhances AP Capacity (V_alt_) under Cd Stress

**Figure 4:**
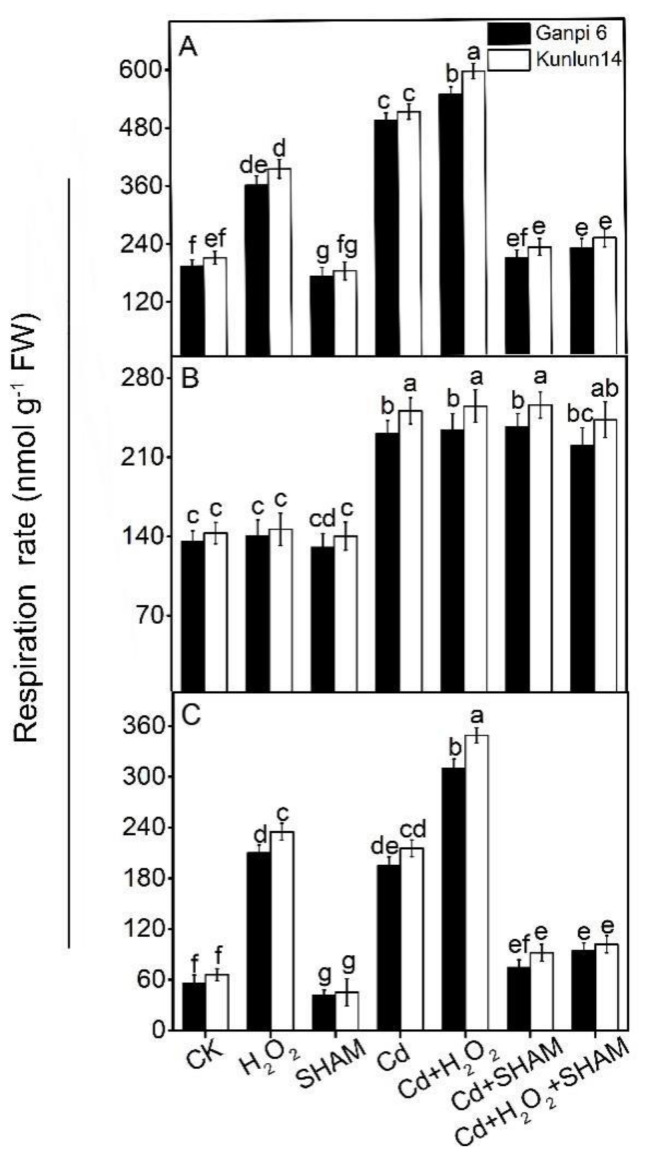
Effects of H_2_O_2_ and SHAM on respiration rates in Ganpi6 and Kunlun14 roots under Cd stress: (**A**) total respiration rate (V_t_); (**B**) cytochrome pathway capacity (V_cyt_); (**C**) alternative pathway capacity (V_alt_). 150 μM Cd, 20 μM H_2_O_2_, and 100 μM SHAM were used (*n* = 3). Different lower case letters represent significant difference at *p* < 0.05.

To further explore the effect of exogenous H_2_O_2_ on respiration under Cd stress, changes in total respiration rate (V_t_), cytochrome pathway capacity (V_cyt_) and V_alt_ were examined. As shown in Figure 4, V_t_ and V_alt_ were significantly increased under H_2_O_2_ treatment alone in Ganpi6 and Kunlun14 roots. Specifically, V_t_ was increased by 2.88× and 3.02×, and V_alt_ was increased by 2.12× and 2.63× in Ganpi6 and Kunlun14, respectively. Under Cd treatment, V_t_, V_alt_ and V_cyt_ were increased by 3.07× and 4.08×, 2.96× and 3.14×, and 1.66× and 1.72× in Ganpi6 and Kunlun14 roots, respectively (Figure 4). Under Cd + H_2_O_2_ treatment, V_alt_ was increased by 50.41% and 58.47% in Ganpi6 and Kunlun14 roots, respectively, compared with Cd treatment alone. In all these treatments, V_cyt_ had almost no changes. When AP was inhibited by SHAM under Cd stress, V_alt_ was decreased to the control level (Figure 4B). Under Cd + H_2_O_2_ + SHAM treatment, V_t_ was markedly reduced. These results indicated that H_2_O_2_-induced respiration is mainly achieved through enhancing V_alt_.

### 3.4. Exogenous H_2_O_2_ Enhances AOX Protein Accumulation under Cd Stress

**Figure 5:**
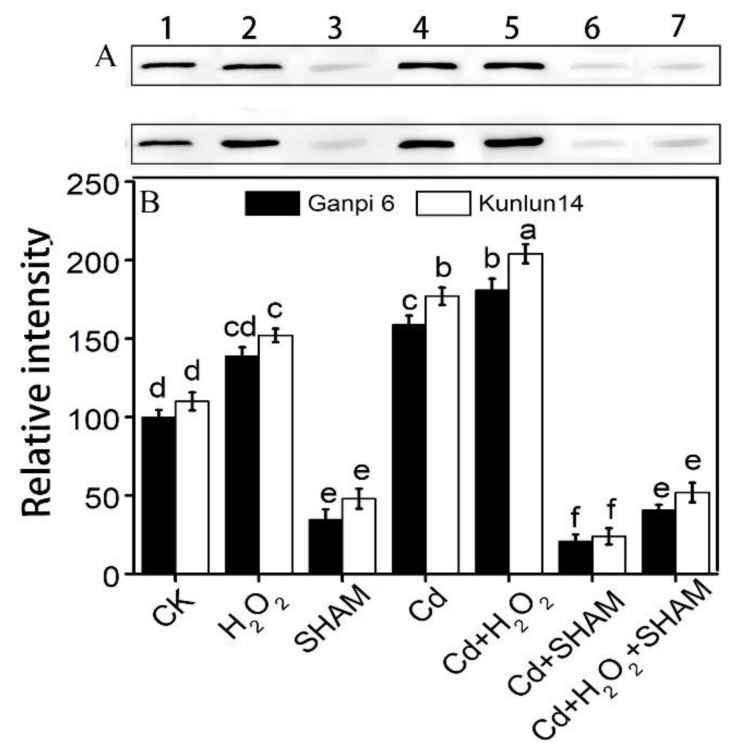
Effects of exogenous H_2_O_2_ on AOX protein level in Ganpi 6 and Kunlun 14 roots under Cd stress: (**A**) Western-blotting analysis of AOX protein; (**B**) quantification of AOX protein. In Figure 5, lane 1: CK; 2: H_2_O_2_; 3: SHAM; 4: Cd; 5: Cd + H_2_O_2_; 6: Cd + SHAM; 7: Cd + H_2_O_2_ + SHAM. AOX protein was quantified by using the ImageJ software (*n* = 3). Different lower case letters represent significant difference at *p* < 0.05.

To further analyze the effect of exogenous H_2_O_2_ on the alternative respiration under Cd stress, the AOX protein level was determined. Western blotting results showed that there was no significant difference in the AOX protein level between Ganpi6 and Kunlun14 under normal condition. Under the exogenous H_2_O_2_ treatment, the AOX protein level had no significant change in Ganpi6; but it was increased by 33.42% in Kunlun14 compared with the control (Figure 5). The AOX protein content was significantly increased by 46.34% and 57.18% in Ganpi6 and Kunlun14 roots, respectively, under Cd stress. H_2_O_2_ treatment further enhanced the AOX protein level in both Ganpi6 and Kunlun14 under Cd stress, with the increase of 31.36% and 42.32%, respectively, in comparison with the Cd stress alone. However, when AP was inhibited by SHAM under Cd + H_2_O_2_ treatment, the AOX protein content was markedly reduced (Figure 5). These results indicated that H_2_O_2_ plays a regulatory role in the Cd-induced AOX protein expression.

### 3.5. AP Is Involved in the Regulation of H_2_O_2_ in Barley Tolerance to Cd Stress

**Figure 6:**
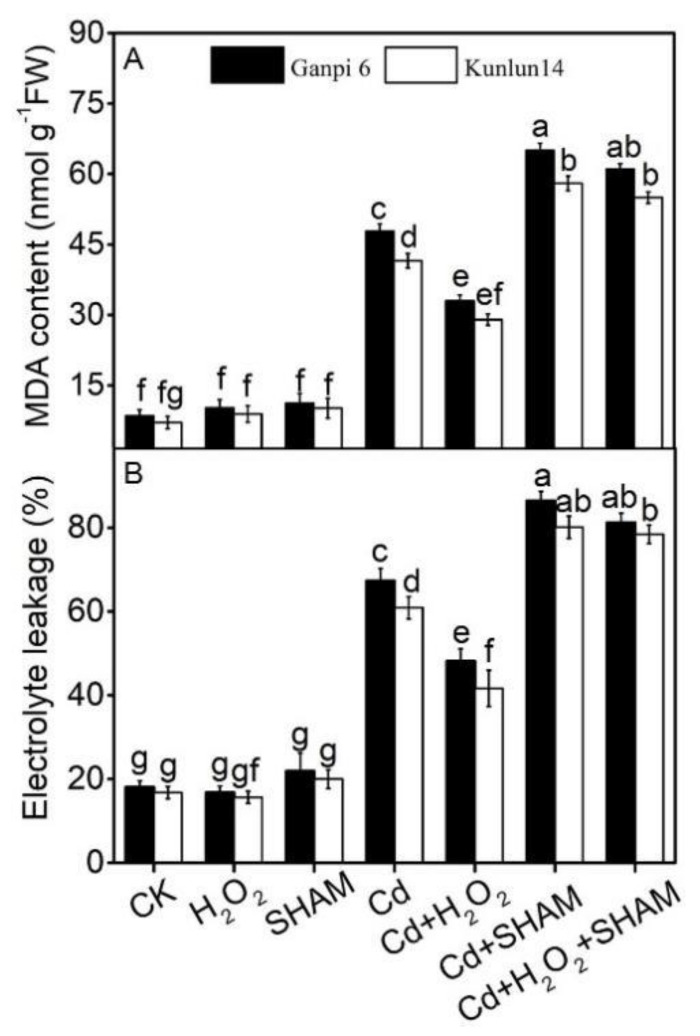
Effects of H_2_O_2_ and SHAM on MDA content (**A**) and electrolyte leakage (EL) (**B**) under Cd stress in Ganpi6 and Kunlun14 roots. Quantities of 150 μM Cd, 20 μM H_2_O_2_, and 100 μM SHAM were used (*n* = 3).Different lower case letters represent significant difference at *p* < 0.05.

AP can improve plant tolerance to environmental stresses [31]. We further tested the relationship between MDA content or EL and AP upon Cd stress. Under Cd stress, in Ganpi6 and Kunlun14 roots, the MDA content was increased by 4.92× and 4.11×, respectively, whereas EL level was increased by 2.74× and 2.13×, respectively. H_2_O_2_ markedly relieved the Cd-induced oxidative stress. MDA content was decreased by 30.96% and 44.77% in Ganpi6 and Kunlun14, respectively, under Cd + H_2_O_2_ treatment in comparison with Cd stress alone; whereas EL was decreased by 28.51% and 41.61%, respectively (Figure 6). When AP was inhibited by SHAM under Cd stress, Cd-induced oxidative damage was intensified. The MDA content was increased by 35.96% and 39.65%; and EL was increased by 28.51% and 31.54%, respectively. Under Cd + H_2_O_2_ + SHAM treatment, in Ganpi6 and Kunlun14 roots, MDA content and EL were increased by 77.14% and 85.71%, respectively, and by 60.27% and 65.11%, respectively, in comparison with Cd + H_2_O_2_ treatment (Figure 6). These results indicated that AP plays an important role in H_2_O_2_ alleviation of the Cd-induced oxidative stress.

### 3.6. Effects of H_2_O_2_ and SHAM on NADH/NAD^+^ and NADPH/NADP^+^ under Cd Stress

**Figure 7:**
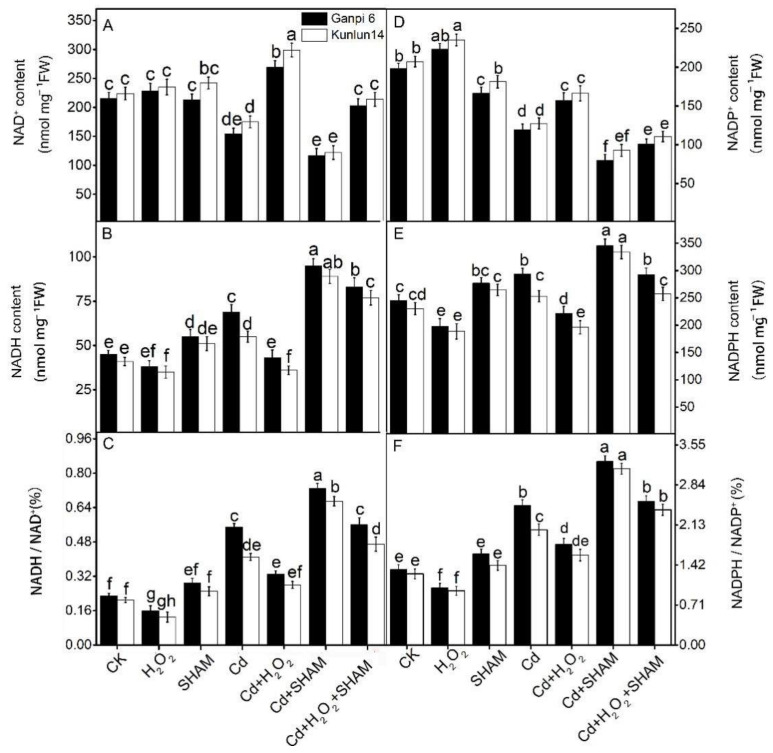
Effects of exogenous H_2_O_2_ and SHAM on NAD^+^ (**A**); NADH (**B**); NADH/NAD^+^ (**C**); NADP^+^ (**D**); NADPH (**E**); NADPH/NADP^+^ (**F**) under Cd stress in Ganpi6 and Kunlun14 roots. 150 μM Cd, 20 μM H_2_O_2_, and 100 μM SHAM were used (*n* = 3). Different lower case letters represent significant difference at *p* < 0.05.

Under stress conditions, AP inhibits the excessive ROS accumulation by consuming excessive reducing power in plants cells [33]. To further explore the effect of AP on the H_2_O_2_-improved Cd tolerance, NADH/NAD^+^ and NADPH/NADP^+^ ratios were examined. Under Cd stress, NAD^+^ and NADP^+^ contents were significantly decreased by 30.31% and 27.83% and by 29.14% and 27.77% in Ganpi 6 and Kunlun 14 roots, respectively. Comparatively, NADH and NADPH contents were increased by 57.61% and 37.62% and by 23.15% and 21.54% in Ganpi 6 and Kunlun 14 roots, respectively. Thus, the NADPH/NADP^+^ ratio and NADH/NAD^+^ ratio were increased by 70.22% and 52.13% and by 127.05% and 69.34% in Ganpi 6 and Kunlun 14 roots, respectively (Figure 7). When AP was inhibited by SHAM under Cd stress, NAD^+^ and NADP^+^ contents were significantly decreased compared with Cd treatment alone; comparatively, NADH and NADPH contents were significantly increased (Figure 7). These results indicated that excessive reducing power was accumulated in barley roots under Cd stress; and AP can utilize the excessive reducing power.

Under Cd stress, exogenous application of H_2_O_2_ resulted in the increase of NAD^+^ and NADP^+^ contents and the decrease of NADH and NADPH contents (Figure 7). In comparison with Cd treatment alone, NAD^+^ and NADP^+^ contents were increased by 12.54% and 29.64% and by 26.13% and 33.34% in Ganpi 6 and Kunlun 14 roots, respectively, under Cd + H_2_O_2_ treatment; while NADH and NADPH contents were decreased by 30.77% and 36.36% and by 18.18% and 24.43%, respectively (Figure 7B,E). Further results showed that the NADPH/NADP^+^ ratio and NADH/NAD^+^ ratio were significantly decreased under Cd+H_2_O_2_ treatment (Figure 7C,F), which were reversed when AP was inhibited by SHAM. These results suggested that AP is involved in the H_2_O_2_-induced Cd tolerance in Ganpi 6 and Kunlun 14 roots, which was more obvious in Kunlun 14.

### 3.7. Effects of Exogenous H_2_O_2_ and SHAM on the AsA-GSH Cycle under Cd Stress

**Figure 8:**
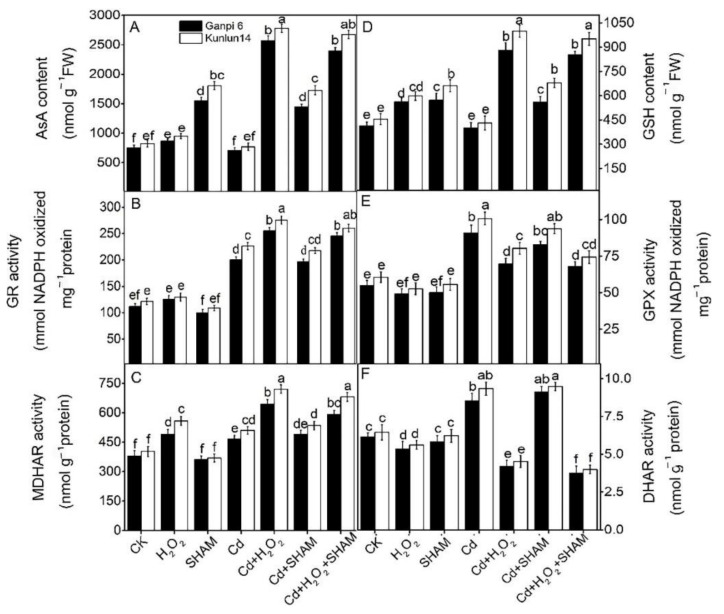
Effects of exogenous H_2_O_2_ and SHAM on AsA (**A**); GR (**B**); MDHAR (**C**); GSH (**D**); GPX (**E**); DHAR (**F**) under Cd stress in Ganpi6 and Kunlun14 roots. 150 μM Cd, 20 μM H_2_O_2_, and 100 μM SHAM were used (*n* = 3). Different lower case letters represent significant difference at *p* < 0.05.

Under stress conditions, plants can remove excessive ROS by increasing antioxidant molecules (such as AsA and GSH) [32]. In order to investigate the effects of H_2_O_2_ and AP on antioxidant molecules under Cd stress, we checked the AsA and GSH contents. Under Cd stress, AsA and GSH contents were significantly increased by 1.14× and 1.59× and by 1.40× and 1.53× in Ganpi 6 and Kunlun 14 roots, respectively (Figure 8A,D). After adding exogenous H_2_O_2_ under Cd stress, AsA and GSH contents were further significantly increased by 38.03% and 48.27% and by 15.91% and 20.11% in Ganpi6 and Kunlun14 roots, respectively, compared to Cd treatment alone (Figure 8A,D). However, when AP was inhibited by SHAM under Cd or Cd + H_2_O_2_ treatment, AsA and GSH contents had no significant changes (Figure 8). This suggested that H_2_O_2_ plays an essential role in regulating ASA and GAH levels under Cd stress. However, AP is not involved in this process.

GSH and AsA regeneration requires enzymes (GR, GPX, MDHAR and DHAR) in the AsA-GSH cycle. Thus, the activities of these enzymes were examined. The results showed that the enzyme activities were significantly increased under Cd stress in Ganpi6 and Kunlun14 roots. GR, MDHR, GPX and DHAR activities increased by 80.68% and 89.16%, by 31.24% and 36.86%, by 63.28% and 67.54%, and by 40.33% and 44.11% in Ganpi6 and Kunlun14 roots, respectively. After adding exogenous H_2_O_2_ under Cd stress, the activities of GR and MDHAR were further significantly increased compared with Cd treatment alone, but the activities of GPX and DHAR were significantly decreased. When AP was inhibited under Cd + H_2_O_2_ treatment, activities of these enzymes had no significant changes compared to Cd + H_2_O_2_ treatment (Figure 8B,C,E,F). These results confirmed that AP is not involved in the H_2_O_2_-induced antioxidant enzyme activities in barley tolerance to Cd stress.

### 3.8. Effects of Exogenous H_2_O_2_ and SHAM on Antioxidant Enzyme Activities under Cd Stress

**Figure 9:**
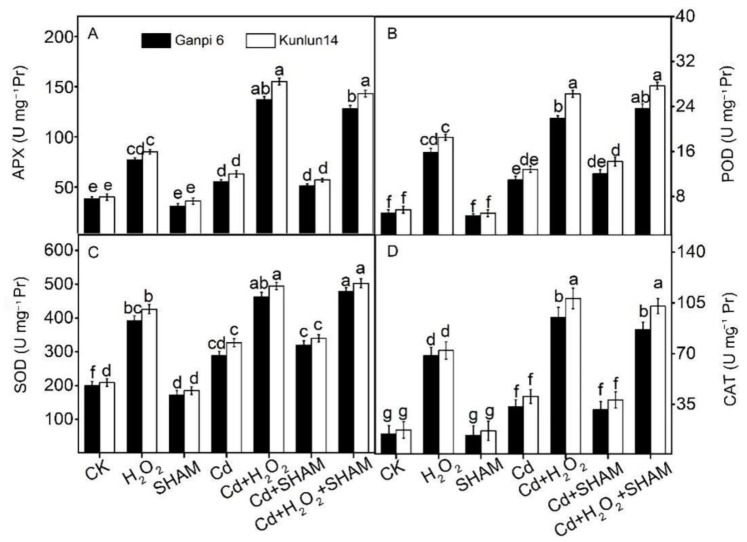
Effects of exogenous H_2_O_2_ and SHAM on APX (**A**); POD (**B**); SOD (**C**); CAT (**D**) under Cd stress in Ganpi6 and Kunlun14 roots. 150 μM Cd, 20 μM H_2_O_2_, and 100 μM SHAM were used (*n* = 3). Different lower case letters represent significant difference at *p* < 0.05.

Under various environmental stresses, plants can eliminate excess ROS by stimulating antioxidant enzymes [31]. As shown in Figure 9, the activities of SOD, CAT, APX and POD were significantly increased under Cd stress. In Ganpi6 and Kunlun14 roots, SOD, CAT, APX and POD activities were increased by 28.61% and 35.84%, by 50.41% and 56.72%, by 16.32% and 22.81%, and by 151.14% and 174.36%, respectively. After adding the exogenous H_2_O_2_ under Cd stress, these antioxidant enzyme activities were further significantly increased compared with Cd treatment alone in Ganpi6 and Kunlun14 roots (Figure 9). When AP was inhibited by SHAM under Cd or Cd + H_2_O_2_ treatment, there was no significant difference in the activities of the four enzymes compared with Cd treatment alone. It indicated that AP is not associated with antioxidant enzyme activities in the process of H_2_O_2_-improved the Cd tolerance.

### 3.9. Effects of Exogenous H_2_O_2_ and SHAM on the Expression of Antioxidant Enzyme Genes under Cd Stress

**Figure 10:**
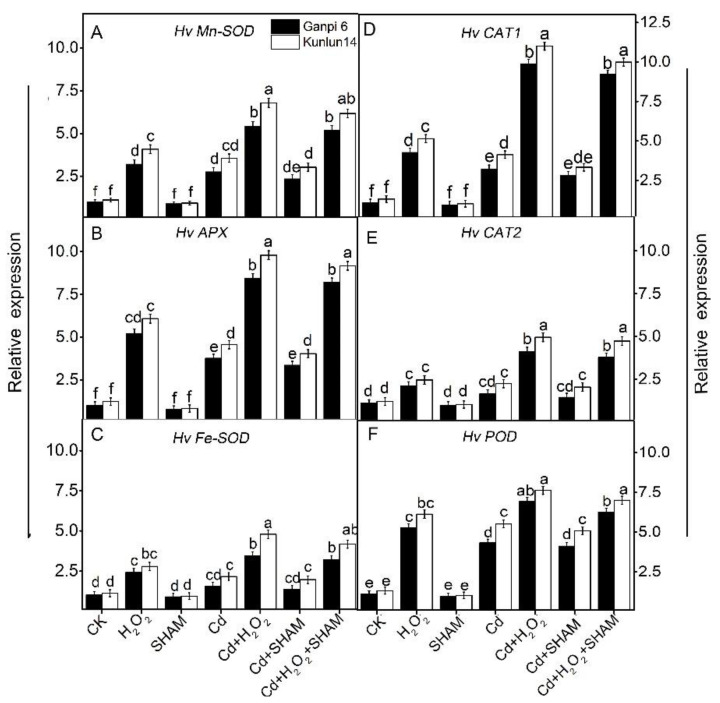
Effects of H_2_O_2_ on the expression of antioxidant enzyme genes in Ganpi6 and Kunlun14 roots under Cd stress. (**A**) *HvMn-SOD*; (**B**) *HvAPX*; (**C**) *HvFe-SOD*; (**D**) *HvCAT1*; (**E**) *HvCAT2*; (**F**) *HvPOD.* 150 μM Cd, 20 μM H_2_O_2_, and 100 μM SHAM were used (*n* = 3). *HvACTIN* was used as the reference gene. Different lower case letters represent significant difference at *p* < 0.05.

To further explore the mechanism of H_2_O_2_-induced enzyme activities, the expressions of antioxidant enzyme genes were analyzed under Cd stress. Cd stress significantly up-regulated the expression of *HvMn-SOD*, *HvAPX*, *HvFe-SOD*, *HvCAT1*, *HvCAT2* and *HvPOD*, which were increased by 1.62× and 2.42×, by 2.73× and 3.42×, by 1.43× and 2.16×, and by 3.98× and 4.69× in Ganpi6 and Kunlun14 roots, respectively. The expression of *Hv-SOD* and *HvCAT2* had no difference compared with that in control. After adding exogenous H_2_O_2_ under Cd stress, the expression of *HvMn-SOD, HvAPX* and *HvCAT1* was increased by 1.32× and 1.51×, by 1.17× and 1.73×, and by 2.32×, 2.98× in comparison with Cd treatment alone in Ganpi6 and Kunlun14 roots, respectively (Figure 10). However, the expression of other genes increased less than 1 time compared with Cd stress. When AP was inhibited under Cd or Cd + H_2_O_2_ treatment, the expression of these antioxidant enzyme genes had no significant changes, further confirming that AP is not associated with the expression of antioxidant enzyme genes in the H_2_O_2_-improved Cd tolerance.

## 4. Discussion

It has been reported that hulless barley showed the higher Cd tolerance than common barley [30]. This study aimed to explore the physiological role of H_2_O_2_ and alternative pathway (AP) in hulless barley response to Cd stress and the relationship between H_2_O_2_ and AP in this process.

H_2_O_2,_ as a signal molecule, plays a central role in plant response to various stresses [10,11,12,26]. In this study, 20 μM H_2_O_2_ markedly counteracted the Cd-induced oxidative stress in barley (Figure 2), indicating that H_2_O_2_ can improve Cd tolerance in Kunlun14. Studies have indicated that AP can improve plant tolerance to heavy metal stresses by inhibiting the accumulation of ROS [23]. Our results showed that Kunlun14 maintains high V_alt_ under Cd stress (Figure 4C) and low oxidative damage (MDA content and EL; Figure 6) compared to Ganpi6. When AP was inhibited by SHAM, MDA content and EL were significantly increased in Kunlun14. This might be due to the dysfunction of AP causing over-reduction of the mitochondrial electron transport chain (mETC), and thus the excessive accumulation of ROS. Therefore, both H_2_O_2_ and AP are involved in Cd tolerance in hulless barley. What is their relationship in hulless barley response to Cd stress? Our results showed that after inhibiting AP under Cd stress, exogenous H_2_O_2_ cannot alleviate the Cd-induced oxidative stress, especially in Kunlun14 (Figure 6), indicating that the functional AP is required in the H_2_O_2_-induced Cd tolerance in Kunlun14.

Under stress conditions, AP consumes the excessive reducing power to prevent oxidative damage, thus enhances stress tolerance in plants [23]. An increased V_alt_ was observed previously in high barley under low-nitrogen stress with decreased reducing power (NADH and NADPH) [20]. Similarly, V_alt_ was significantly higher in Kunlun14 than that in Ganpi6 (Figure 4C), while reducing power (NADH and NADPH) and oxidative damage indices (MDA and EL) were significantly lower in Kunlun14 (Figure 7A,D) under Cd stress. After applying H_2_O_2_ under Cd stress, V_alt_ was further significantly increased, however, NADH and NADPH contents were reduced (Figure 7A,D). When AP was inhibited by SHAM under Cd + H_2_O_2_ treatment, NADH and NADPH contents were increased more in Kunlun14 than in Cd + H_2_O_2_ treatment alone (Figure 7A,D), indicating that H_2_O_2_ can promote AP to remove more reducing power to alleviate the Cd-induced oxidative damage. 

Studies have shown that H_2_O_2_ can induce a significant increase of AP under environmental stresses [26]. It was reported that exogenous H_2_O_2_ induces the expression of *AOX1* in *Petunia hybrida* under low temperature stress, and the AP capacity was also enhanced [34]. Application of exogenous H_2_O_2_ significantly increased the AP capacity and AOX protein content in petunia suspension cells [34]. Another study showed that application of exogenous H_2_O_2_ for 20 min under water stress, the AP capacity and the expression of *AOX1* family genes in wheat leaves were significantly increased [35]. Similar results were also observed in our observations. Exogenous H_2_O_2_ promoted more increase of *HvAOX1a* expression (Figure 3A)*,* V_alt_ (Figure 4C) and AOX protein (Figure 5) under Cd stress in Kunlun14 than in Ganpi6. Taken together, these results indicate that H_2_O_2_ can promote AP to remove more reducing power at AOX transcription, AOX protein, and AP capacity, thus enhancing the Cd tolerance in Kunlun14.

Antioxidant defense systems have been widely proven to be the core factor in plant defense against oxidative stresses [29,30,31,32]. Exogenous H_2_O_2_ significantly increased the AsA and GSH content in Kunlun14 and Ganpi6, which exhibited a similar pattern to a previous study in different species [13,14,21]. GR, GPX, MDHAR and DHAR are four key enzymes in the AsA-GSH cycle. GR catalyzes the reaction converting GSSG to GSH [27]. Both the GSH content and the GSH:GSSG ratio were all increased when GR was over-expressed in *E. coli* [36]. Application of exogenous H_2_O_2_ raised the GR activity, which resulted in increased GSH content in Cd-treated wheat seedlings [36]. As observed in our study, exogenous H_2_O_2_ also increased the GR activity, but decreased the GPX activity under Cd stress (Figure 8B,E). This might be the main reason for the high level of GSH content under Cd + H_2_O_2_ treatment (Figure 8D). MDHAR and DHAR are two key enzymes in the AsA-GSH cycle, which maintain the homeostasis of AsA [28]. In wheat leaves, drought stress markedly increased the AsA content and the activities of MDHAR and DHHAR, which were further enhanced in the presence of H_2_O_2_ [36]. However, our observations showed that exogenous H_2_O_2_ increased the MDHAR activity, but decreased the DHAR activity (Figure 8C,F), which was inconsistent with previous study. This might be the main reason for the high AsA content under Cd + H_2_O_2_ treatment.

Increasing findings have shown that the antioxidant enzyme activities are significantly increased under various stress conditions [30,31,32,33]. Similarly, in our results, these antioxidant enzyme activities were markedly elevated in Kunlun14 roots under Cd stress (Figure 9), and they were further enhanced in the presence of H_2_O_2_. It was consistent with the previous study in *Petunia hybrida* [34]. In addition, antioxidant enzyme genes (*HvMnSOD, HvCAT1, HvAPX)* were significantly up-regulated (Figure 9), similar as observed in previous study in *Medicago truncatula* [29]. Furtherly, the Cd-induced oxidative stress was much relieved in Kunlun14 under Cd + H_2_O_2_ treatment (Figure 6). This indicated that antioxidant defense systems play an important role in the H_2_O_2_-enhanced defense against oxidative stress in barley.

AP plays an important role in maintaining ROS homeostasis [32]. In addition, some studies have pointed out that AOX deficiency would lead to the increase of ROS-related scavenging enzyme activities [35]. Studies have shown that exogenous H_2_O_2_ can enhance the tolerance of *mangrove* to Cd stress by synergistic elimination of ROS through promoting AP and antioxidant enzyme activities [8]. The other report indicated that the exogenous H_2_O_2_ improved the tolerance of wheat to drought stress by jointly improving antioxidant enzymes and AP [33]. In this study, when AP was inhibited under Cd + H_2_O_2_ treatment, antioxidant enzyme activities and antioxidant molecule contents had no significant changes, suggesting that AP is not involved in the H_2_O_2_-induced antioxidant defense system in highland barley tolerance to Cd stress. These results are not consistent with the previous studies [8,33], which might be ascribed to the differences in materials, stress conditions and treatment concentrations.

## 5. Conclusions

Above results clarified the physiological and molecular mechanisms of AP in H_2_O_2_-enhanced hulless barley Cd tolerance. Exogenous H_2_O_2_ promoted the expression of *HvAOX1a* (5.37×), *HvAOX1d1* (1.87×), and *HvAOX1d2* (1.06×), AOX proteins (42.32%) and V_alt_ (58.47%) compared with Cd stress. H_2_O_2_ prevented the over-accumulation of ROS by enhancing AP to dissipating excess NADH/NAD^+^ (46.50%) and NADPH/NADP^+^ (40.21%) ratios, ROS-related scavenging enzymes and antioxidant molecules. AP had no correlation with antioxidant defense system in H_2_O_2_-related hulless barley tolerance to Cd stress. In this study, we confirmed that AP plays a pivotal role in H_2_O_2_-elevated Cd tolerance in hulless barley.

## Figures and Tables

**Table 1:** Primer Sequences.

Primer Name	Primer Sequence (5’ to 3’)
qHvAOX1a-F	GCAACGAACCTACAAGCGTG
qHvAOX1a-R	AAGAGCCCAGCACCAACAA
qHvAOX1d1-F	CCTCCCATTAGCTTTTCGACCAG
qHvAOX1d1-R	CGGTAGCACGTAACAGCGTGGACT
qHvAOX1d2-F	TACGACCACGAGTTTCGCGAGCA
qHvAOX1d2-R	GCTAAAGAGCCCTCATTTCCTC
HvMnSOD-F	CAGGTCGTACAACWCGATTA
HvMnSOD-R	CGTCAAGAAATCCAAACAGTC
HvFeSOD-F	GCAACGTTGGTACAACGGA
HvFeSOD-R	CGTAAAGAGCGTCATTTGG
HvPOD-F	GGTCCCATTACCTTTTCGTGGTC
HvPOD-R	GCCTAGCACGTAACACGCTGACT
HvCAT1-F	TAGCAGGACGAGTAACGCCTGGT
HvCAT1-R	CGTAAAGAGCCCTCTAATCG
HvCAT2-F	GCAACGAACCTACAACCGTC
HvCAT2-R	AAGAGCCCAGCACCAACAAT
HvAPX-F	GCTCCCATTAGCTTTTCGACAC
HvAPX-R	GCCTAGCACGTAACAGCGTTCA
HvACTIN-F	GTGGTCGTACAACWGGTATTGTG
HvACTIN-R	GCTCATCAAATCCAAACACTG
